# Missense Mutations in Desmoplakin Plakin Repeat Domains Have Dramatic Effects on Domain Structure and Function

**DOI:** 10.3390/ijms23010529

**Published:** 2022-01-04

**Authors:** Fiyaz Mohammed, Elena Odintsova, Martyn Chidgey

**Affiliations:** 1 Institute of Immunology and Immunotherapy, University of Birmingham, Birmingham B15 2TT, UK; f.mohammed@bham.ac.uk; 2 Institute of Cancer and Genomic Sciences, University of Birmingham, Birmingham B15 2TT, UK; e.odintsova@bham.ac.uk

**Keywords:** desmosome, desmoplakin, plakin repeat domain, mutation, arrhythmogenic right ventricular cardiomyopathy

## Abstract

Plakin repeat domains (PRDs) are globular modules that mediate the interaction of plakin proteins with the intermediate filament (IF) cytoskeleton. These associations are vital for maintaining tissue integrity in cardiac muscle and epithelial tissues. PRDs are subject to mutations that give rise to cardiomyopathies such as arrhythmogenic right ventricular cardiomyopathy, characterised by ventricular arrhythmias and associated with an increased risk of sudden heart failure, and skin blistering diseases. Herein, we have examined the functional and structural effects of 12 disease-linked missense mutations, identified from the human gene mutation database, on the PRDs of the desmosomal protein desmoplakin. Five mutations (G2056R and E2193K in PRD-A, G2338R and G2375R in PRD-B and G2647D in PRD-C) rendered their respective PRD proteins either fully or partially insoluble following expression in bacterial cells. Each of the residues affected are conserved across plakin family members, inferring a crucial role in maintaining the structural integrity of the PRD. In transfected HeLa cells, the mutation G2375R adversely affected the targeting of a desmoplakin C-terminal construct containing all three PRDs to vimentin IFs. The deletion of PRD-B and PRD-C from the construct compromised its targeting to vimentin. Bioinformatic and structural modelling approaches provided multiple mechanisms by which the disease-causing mutations could potentially destabilise PRD structure and compromise cytoskeletal linkages. Overall, our data highlight potential molecular mechanisms underlying pathogenic missense mutations and could pave the way for informing novel curative interventions targeting cardiomyopathies and skin blistering disorders.

## 1. Introduction

**Figure 1:**
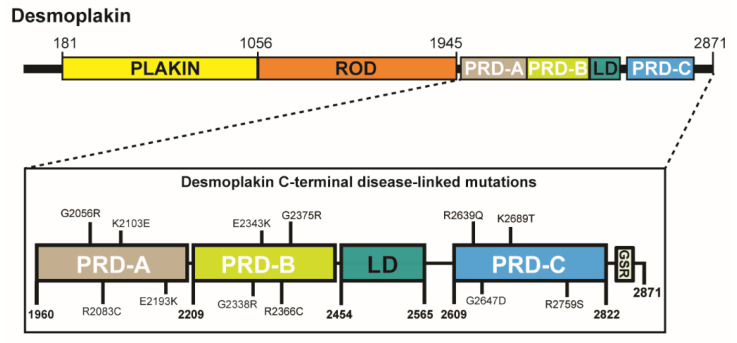
Domain architecture of desmoplakin. The protein encompasses an N-terminal plakin domain, a central rod domain that forms a helical coiled-coil followed by a C-terminal tail region comprising three plakin repeat domains (PRDs) and a linker domain (LD). The position of disease-linked mutations introduced into PRDs A, B and C are indicated. GSR, glycine-serine-arginine-rich region. The domain boundaries are indicated.

Desmoplakin is a cytoplasmic desmosomal protein that plays a central role in intercellular adhesion [1]. It is a member of the plakin family of cytolinkers [2] and is of crucial importance for a normal desmosomal structure and function. Severance of the link that desmoplakin provides between other desmosomal proteins and the intermediate filament (IF) cytoskeleton can have catastrophic effects on tissue integrity, both in transgenic mice [3] and humans [4]. Desmoplakin has a tripartite structure that encompasses a globular N-terminal head domain, a central alpha-helical rod element that mediates dimerization, and a C-terminal tail module. The head region associates with desmosomal proteins, such as plakoglobin and the plakophilins, whereas the tail section mediates interactions with IF proteins, including keratin, desmin and vimentin, through three homologous plakin repeat domains (PRDs) and a linker module (Figure 1).

PRDs are found in a number of plakin proteins including desmoplakin, envoplakin, plectin, bullous pemphigoid antigen 1, microtubule-actin cross-linking factor 1 and epiplakin [5]. The desmoplakin PRDs are of three types based on their sequence homology, and designated PRD-A, PRD-B and PRD-C. All contain 4.5 copies of the canonical 38-amino acid plakin repeat (PR) motif, which includes an 11 residue β-hairpin followed by an antiparallel pair of α-helices. The crystal structures of desmoplakin PRDs show a similar fold characterised by a conserved positively charged groove on their surface [6,7]. A mechanism whereby dimeric IF rods slot into PRD grooves with the interface stabilised by electrostatic interactions between basic residues in the groove and acidic side chains on IF rods has been proposed [8]. In addition to its three PRDs, desmoplakin possesses a linker module, located between PRDs B and C. Linker modules encompass two PR-like motifs and are found in several other plakin proteins [5]. The crystal structure of periplakin linker shows an elongated bi-lobed domain that is transected by an electropositive groove, and modelling suggests that the desmoplakin linker exhibits a similar overall topology [7,9]. Basic linker domain side chains recognise acidic IF rod residues by electrostatic complementary in a similar mechanism to that demonstrated by PRDs [9]. Crystallography and small angle X-ray scattering analysis suggest that the three desmoplakin PRDs and linker form an elongated ‘beads on a string’ structure [7]. The relative orientation of grooves in PRDs A and B [7] suggest that tandem PRDs are unlikely to bind to the same IF dimer. Instead, we have proposed that the PRDs are likely to engage adjacent dimeric IF rods clustering them together within a fully assembled IF [5].

Interactions between the desmoplakin tail domain and the IF cytoskeleton are vital for maintaining tissue integrity. The loss of this interaction due to truncating mutations causes lethal acantholytic epidermolysis bullosa, characterised by catastrophic skin blistering and early death [4]. Numerous other disease-associated mutations have been documented throughout the desmoplakin (*DSP*) gene [10] resulting in inherited diseases that affect the heart and skin. Arrhythmogenic right ventricular cardiomyopathy (ARVC) is one such disease characterised by life-threatening arrhythmias and associated with increased risk of sudden heart failure [11]. It arises from mutations in all five desmosomal genes that are expressed in the heart, including *DSP*. Although disease-linked missense mutations have been identified in the desmoplakin C-terminal PRDs [10], the mechanisms by which such substitutions drive pathogenicity remain elusive. Herein, we show that the introduction of disease-linked single point mutations within discrete desmoplakin PRDs can exert destabilising effects leading to the insolubility of recombinant PRDs expressed in *E. coli* cells. We demonstrate that a disease-associated variant in desmoplakin PRD-B disrupts co-localization with vimentin IFs in transfected HeLa cells and establish the importance of PRDs B and C in targeting desmoplakin to the IF cytoskeleton. We use in silico tools to predict the effects of our mutations on PRD function and compare the results with our bacterial expression data. Finally, molecular modelling approaches are exploited to examine the effects of the substitutions on PRD structure.

## 2. Results

### 2.1. Mutations G2056R and E2193K in PRD-A, G2338R and G2375R in PRD-B and G2647D in PRD-C Render the Domains Insoluble Following Expression in Bacterial Cells

**Figure 2:**
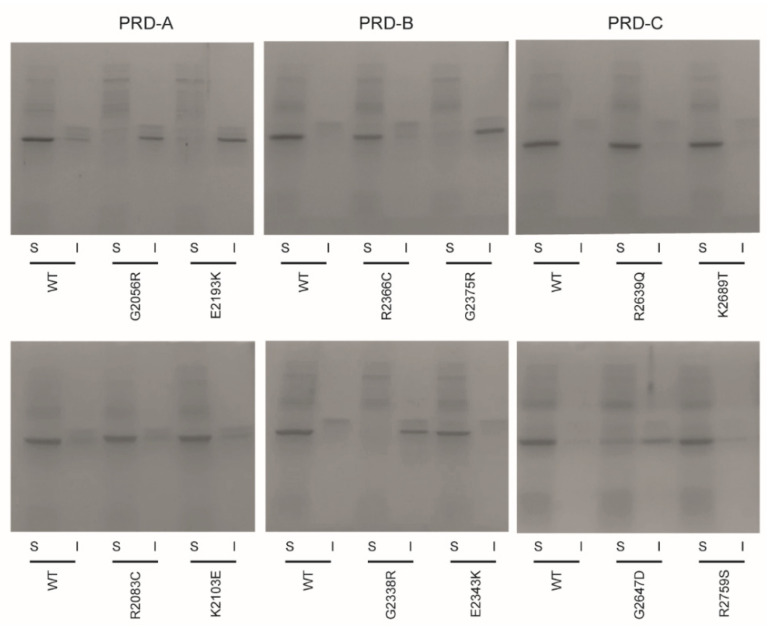
Disease-associated mutations affect the solubility of desmoplakin PRDs expressed in bacterial cells. Protein expression was induced with isopropyl-β-D-thiogalactopyranoside (IPTG) and soluble (S) and insoluble (I) fractions analysed using SDS-PAGE. All three wild-type (WT) PRDs are expressed in soluble form. Introduction of mutations G2056R and E2193K in PRD-A and G2338R and G2375R in PRD-B rendered these proteins insoluble (protein sequestered in inclusion bodies). Incorporation of the G2647D variant in PRD-C led to partially insoluble protein. The remaining mutations exhibited no adverse effect on PRD solubility.

In order to examine the effect of ARVC mutations on PRD stability we cloned DNA encoding desmoplakin PRD-A (residues 1960-2208), PRD-B (residues 2209-2456) and PRD-C (residues 2609-2822) into vector pProExHTc. Bacterial cells were transformed with vector DNA and expression of recombinant protein induced with isopropyl-β-D-thiogalactopyranoside (IPTG). SDS-PAGE analysis of total cell lysates revealed intense bands corresponding to the molecular weight of desmoplakin PRDs-A, B and C (~27 kDa) (Figure S1). In the subsequent experiments, the cells were subjected to lysis by sonication following induction with IPTG and the crude cell extract centrifuged to yield supernatant (soluble) and pellet (insoluble) fractions. All three wild-type PRDs were detected in the soluble fraction of the *E. coli* cells (Figure 2), indicative of a correctly folded protein. A number of disease-causing mutations in *DSP* were identified using the Human Gene Mutation Database (HGMD; http://www.hgmd.cf.ac.uk/ac/index.php, accessed 1 June 2020). Twelve missense mutations were selected and introduced into DNA encoding desmoplakin PRD-A (G2056R, R2083C, K2103E and E2193K), PRD-B (G2338R, E2343K, R2366C and G2375R) and PRD-C (R2639Q, G2647D, K2689T and R2759S) (Figure 1). For 7 of the 12 variants (R2083C, K2103E, E2343K, R2366C, R2639Q, K2689T and R2759S) prominent bands corresponding to the PRD protein was only observed in the supernatant lanes, indicating that the mutant PRD protein was soluble in each case (Figure 2). By contrast, for 4 of the 12 variants, intense bands were solely detected in the pellet lanes, highlighting that the PRD proteins were insoluble in each case. Of these, two were located in PRD-A (G2056R and E2193K) and two in PRD-B (G2338R and G2375R). Finally, for the PRD-C^G2647D^ variant, bands were observed in both the supernatant and pellet lanes, indicative of a partially unfolded PRD-C protein as a consequence of the mutation (Figure 2).

### 2.2. Predicting the Effect of Mutations on PRD Structure and Function

**Figure 3:**
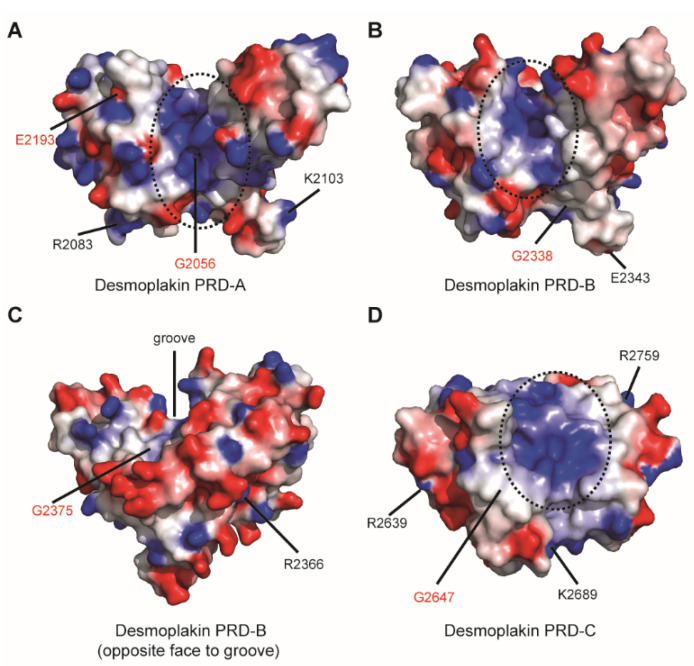
Molecular surface representation of desmoplakin PRDs (**A**) (PDB: 5DZZ), (**B**,**C**) (PDB: 1LM7) and (**D**) (PDB: 1LM5) showing the electrostatic potential. Negative potential is highlighted in red and positive potential in blue. Surface potential was calculated with DelPhi with the potential scale ranging from −7 to +7 in units of kT/e. The disease-linked variants investigated in this study are mapped. The location of the basic groove is shown (black dashed circle). Mutation of residues that rendered PRDs insoluble following recombinant expression in bacterial cells are highlighted in red.

All three desmoplakin PRDs display a large positively charged groove lined with conserved amino acids [6,7] (Figure 3). PRD groove basic residues recognise acidic side chains in IF rods via electrostatic complementarity, allowing IF rods to slot into the grooves [5,8]. The mapping of disease-causing mutations in each desmoplakin PRD shows that the majority of the affected residues, with the exception of G2056, are distal to the putative IF binding basic groove (Figure 3). Strikingly, those residues that when mutated rendered their PRD entirely insoluble in bacteria (G2056, E2193, G2338, G2375) were highly invariant (i.e., with a consistency score ≥ 7), as calculated using the PRALINE multiple sequence alignment toolkit. Residue G2647, which rendered PRD-C partially insoluble, was less well conserved (consistency score 6). Similarly, the residues that yielded a comparable soluble expression profile to wild-type protein when mutated were less well conserved (i.e., with a consistency score ≤ 6) (Table 1; Figure S2). A variety of in silico bioinformatics tools were used to predict whether disease-associated amino acid substitutions affect the desmoplakin PRD function. SIFT (Sorting Intolerant from Tolerant) is one such tool that exploits sequence homology data [12]. SIFT analysis highlighted that 6 of the 12 disease-linked mutations (G2056R, R2083C, G2338R, R2366C, G2375R and G2647D) are likely to compromise desmoplakin PRD function (Table 1). The results are in reasonable agreement with our bacterial recombinant expression of *DSP* variants, with the exception of mutations R2083C and R2366C, which were predicted by SIFT to be deleterious to PRD function despite being expressed in soluble form in bacterial cells. In addition, E2193K was predicted to be tolerated with no adverse functional effects but was expressed as insoluble aggregates in bacterial cells. Missense3D is an interactive tool that exploits structural protein information to predict the structural changes introduced by single amino acid substitutions [13]. The Missense3D server predicted that 4 of the 12 mutations (G2056R, G2338R, G2375R and G2647D) would exert structural vulnerabilities within the PRD, whilst the remaining substitutions are likely to be tolerated (Table 1). The predictions derived from Missense3D are broadly consistent with our bacterial expression studies, with the exception of mutation E2193K. In the latter case, the PRD-A variant was expressed as insoluble aggregates in *E. coli*, despite the server predicting no adverse effects on the native conformation.

We further assessed the impact of disease-linked single point substitutions on desmoplakin’s PRD function using the in silico tools PolyPhen-2 [14], DynaMut [15], ENCoM [16], mCSM [17], SDM [18] and DUET [19]. These interactive packages exploit different resources to classify whether mutations are likely to adversely affect protein structure and function. PolyPhen-2 is a sequence-based tool that combines homology information with structural data, to predict the possible impact of amino acid substitutions. PolyPhen-2 predicted all of the mutations to be either possibly damaging or probably damaging (Table S1). Other structure-based protein stability predictors such as DynaMut, ENCoM mCSM, SDM and DUET also calculate the stability changes (ΔΔG in kcal/mol) caused by single residue substitutions. None of these showed a good correlation to our bacterial expression data. DynaMut, ENCoM mCSM, SDM and DUET predicted that 5, 9, 10, 11 and 10 of the 12 mutations would be destabilising, respectively (Table S1). All five of the mutations that rendered each PRD insoluble following bacterial expression were predicted as destabilising by mCSM, SDM and DUET, which is perhaps not surprisingly given the high proportion of mutants that were assigned as structurally debilitating.

### 2.3. Mutation G2375R Adversely Affects Targeting to the Intermediate Filament Cytoskeleton in Transfected HeLa Cells

**Figure 4:**
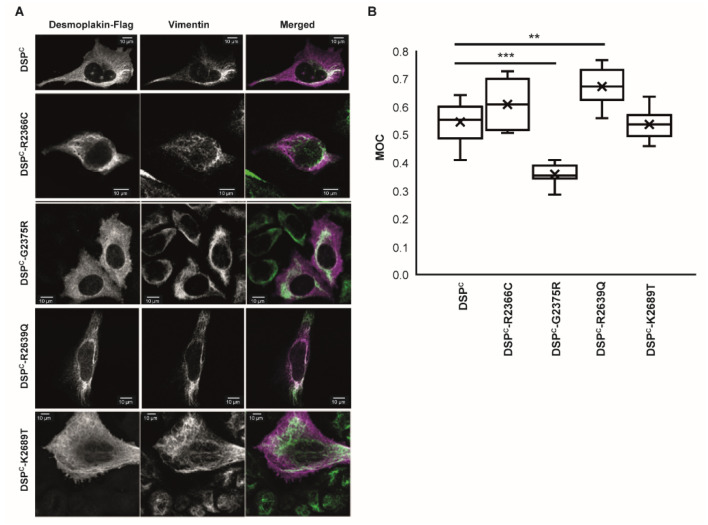
Mutation G2375R compromises co-localisation of the DSP^C^ protein with vimentin intermediate filaments in transfected HeLa cells. (**A**) Constructs encoding human desmoplakin residues 1960-2822 with a C-terminal FLAG tag were transfected into HeLa cells. Representative images of cells stained with anti-FLAG and anti-vimentin antibodies are shown. (**B**) Tukey plot showing Manders’ overlap coefficient (MOC) for each image with the median, 25th and 75th percentiles of each distribution. The cross represents the mean of the data set. At least five fields of view were analysed for each experiment. z-stacks were taken for each field and overlap coefficients calculated for each individual z-stack. Each experiment was repeated two to three times. A two-tail *t* test was performed on the data. *** *p* = 0.0001; ** *p* = 0.003.

Six desmoplakin PRDs harbouring disease-linked mutations were selected for co-localisation analysis with IFs in HeLa cells using immunofluorescence microscopy; two from PRD-A, two from PRD-B and two from PRD-C. Of these, three were soluble (R2366C, R2639Q and K2689T) and three were insoluble (G2056R, E2193K and G2375R) based on their recombinant expression profiles in *E. coli* (Figure 2). Individual PRDs are poorly expressed in transfected HeLa cells, necessitating the use of a longer C-terminal DSP^C^ construct consisting of desmoplakin residues T1960-A2822, encompassing all three PRDs and the linker module (but excluding the glycine-serine-arginine-rich (GSR) region) (Figure S3A). This construct was previously shown to co-localise with IFs in transfected HeLa cells [8,9]. A band corresponding to the expected molecular weight of the DSP^C^ construct (~95kDa) was observed using Western blotting (Figure S3B). DSP^C^ proteins containing mutations G2056R, E2193K and G2375R were very poorly expressed relative to the wild-type DSP^C^ protein. The expression of R2369Q was slightly improved, although still less than that of wild-type DSP^C^. By contrast, the DSP^C^ proteins encompassing R2366C and K2689T variants were expressed at comparable levels, perhaps somewhat better, than the wild-type (Figure S3B). As expected, the wild-type DSP^C^ construct showed extensive co-localisation with vimentin IFs (Figure 4A). The DSP^C^ constructs incorporating soluble mutations R2366C, R2639Q and K2689T showed similar patterns of staining to the wild-type DSP^C^ protein with extensive co-localisation with the IF cytoskeleton. By contrast, the DSP^C^-G2375R construct showed a diffuse staining pattern throughout the cytoplasm (Figure 4A). It was not possible to perform immunofluorescence co-localisation analysis of DSP^C^-G2056R and DSP^C^-E2193K variants with vimentin, because of their very poor expression in HeLa cells (Figure S3B). We used Manders’ method [31], which measures the fraction of pixels with positive values in two channels, to quantify the levels of co-localisation between the DSP^C^ proteins and vimentin in transfected HeLa cells. The value of Manders’ overlap coefficient (MOC) ranges from 0 to 1 with an MOC of 0.5, implying that DSP^C^ in one channel co-localises with 50% of the vimentin in another channel. The wild-type DSP^C^ exhibited an MOC of 0.52, indicating substantial co-localisation with vimentin, in agreement with our previous results [9] (Figure 4B). The MOC values of DSP^C^ encompassing soluble mutations R2366C, R2639Q and K2689T were not significantly different to that of the wild-type protein. By contrast, the DSP^C^ protein incorporating insoluble mutation G2375R had a reduced MOC value of 0.35, indicating a reduced co-localisation with the IF cytoskeleton (Figure 4B).

### 2.4. Deletion of Either PRD-B or PRD-C Adversely Affects Targeting of DSP^C^ to IFs

**Figure 5:**
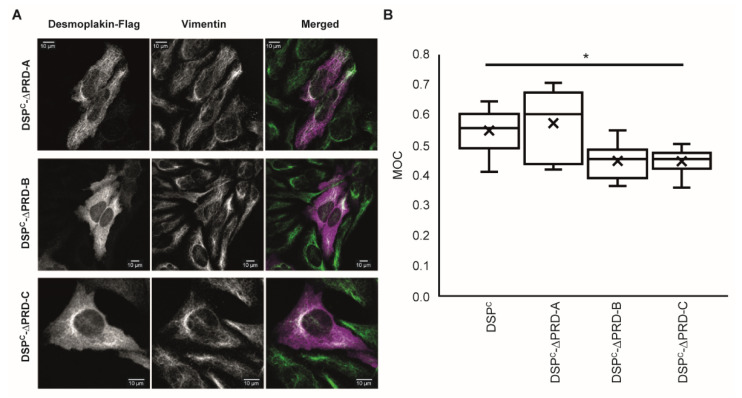
Deletion of PRDs B and C compromise the ability of the DSP^C^ protein to co-localise with vimentin intermediate filaments in transfected HeLa cells. (**A**) Constructs encoding desmoplakin residues 2209-2822 (DSP^C^ΔPRD-A), 1960-2208 fused to 2454-2822 (DSP^C^ΔPRD-B) and 1960-2608 (DSP^C^ΔPRD-C) (each with a C-terminal FLAG tag) were transfected into HeLa cells. Representative images of cells were stained with anti-FLAG and anti-vimentin antibodies are shown. (**B**) Tukey plot showing MOC for each image with the median, 25th and 75th percentiles of each distribution. The cross represents the mean of the data set. At least five fields of view were analysed for each experiment. z-stacks were taken for each field and overlap coefficients calculated for each individual z-stack. Each experiment was repeated two to three times. A two-tail t test was performed on the data. * *p* = 0.05.

All three desmoplakin PRDs possess a basic groove and it seems likely that each is able to bind to IF proteins independently [6,7]. To further investigate the role of each desmoplakin PRD in IF binding, we produced truncated DSP^C^ proteins lacking PRD-A (DSP^C^ΔPRD-A), PRD-B (DSP^C^ΔPRD-B) and PRD-C (DSP^C^ΔPRD-C). The three deletion proteins demonstrated the expected molecular weight (~68kDa) using Western blotting following expression in HeLa cells (Figure S3C). Proteins DSP^C^ΔPRD-A and DSP^C^ΔPRD-B were poorly expressed compared to wild-type DSP^C^ (Figure S3C). When transfected into HeLa cells, DSP^C^ΔPRD-A showed a similar pattern of staining to wild-type DSP^C^, with no significant change in the MOC value (Figure 5A,B). The DSP^C^ΔPRD-B and DSP^C^ΔPRD-C proteins showed an irregular staining pattern with dense dot-like structures, predominantly in the perinuclear area. In both cases, the MOC value was lower than wild-type (0.44 versus 0.52), but the difference in the MOC values did not achieve statistical significance, despite the marked differences in the staining patterns. This may be partly attributed to the diffuse cytosolic distribution of the delocalised proteins (Figure 5A,B). Overall, the data suggest that PRD-A is dispensable with respect to targeting IFs and establishes the importance of PRD-B and PRD-C in maintaining stable cytoskeletal linkages.

### 2.5. Modelling the Effect of Mutations on PRD Structure

**Figure 6:**
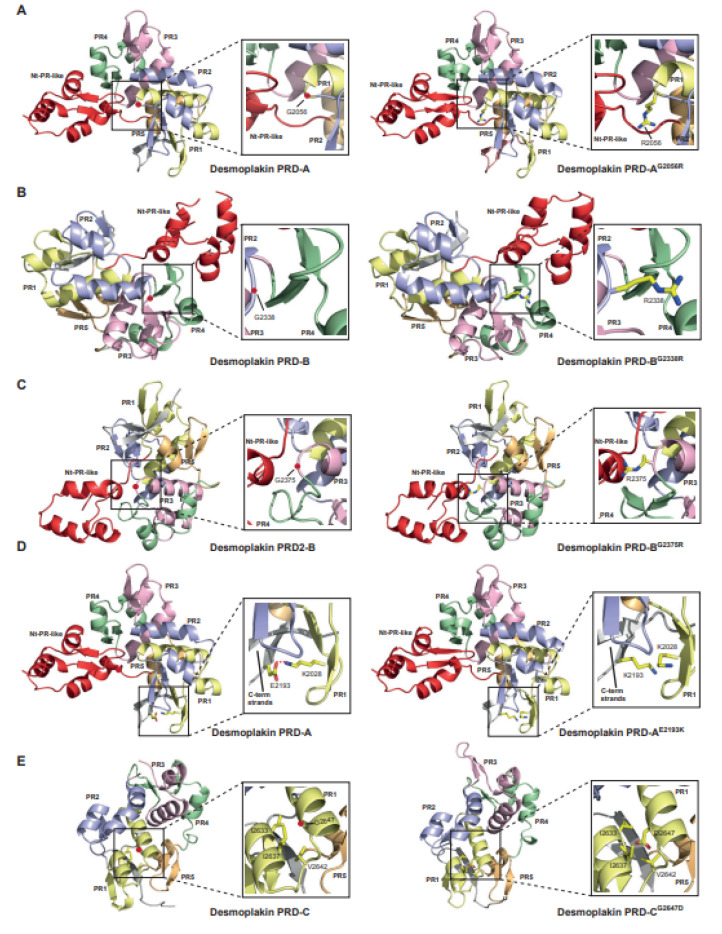
Examining the effects of damaging disease-associated mutations on PRD structure. Damaging mutations that lead to insoluble expression in bacterial cells were modelled. (**A**) Analysing the impact of the disease-related variant G2056R on desmoplakin PRD-A. Ribbon diagram of the desmoplakin PRD-A crystal structure shows that G2056 (red sphere) contributes to a sharp turn in PR1 (left panel). Introduction of R2056 leads to substantial clashes with the Nt-PR-like motif (right panel). (**B**) Assessing the effect of disease-associated variant G2338R on desmoplakin PRD-B. Ribbon diagram of the desmoplakin PRD-B crystal structure shows that G2338 (red sphere) contributes to a sharp turn in PR2 (left panel). Introduction of R2338 leads to substantial clashes with PR4 (right panel). (**C**) Probing the impact of the disease-related variant G2375R on desmoplakin PRD-B. Ribbon diagram of the desmoplakin PRD-B crystal structure shows that G2375 (red sphere) contributes to a sharp turn in PR3 (left panel). Introduction of R2375 leads to substantial clashes with the Nt-PR-like motif (right panel). (**D**) Assessing the effect of ARVC-linked variant E2193K on desmoplakin PRD-A. Ribbon diagram of the desmoplakin PRD-A crystal structure highlighting the electrostatic interaction (red dashed line) between E2193 and K2028 (left panel). Introduction of K2193 leads to a loss of this interaction and charge repulsion effects with K2028 (right panel). (**E**) Investigating the effect of ARVC-linked variant G2647D on desmoplakin PRD-C. Ribbon diagram of the desmoplakin PRD-C crystal structure highlights that G2647 (red sphere) is found at the helical interface of PR1 (left panel). Introduction of D2647 leads to a negative charge in a non-polar environment (right panel). Plakin repeats 1–5 are yellow, blue, pink, green and orange, respectively, the Nt-PR-like domain is highlighted in red and non-repeat regions are shown in grey. Boxes show close up views of the relevant interactions.

To further investigate the mechanisms by which single-point mutations contribute to disease pathogenesis, we modelled the effect of these substitutions on desmoplakin’s PRD structure (Figure 6). We identified multiple mechanisms by which disease-causing mutations could potentially destabilise the PRD structure and compromise the tethering function. Three of the disease-linked mutations involve non-conservative substitutions in which a smaller glycine residue is replaced by a considerably larger positively charged arginine (G2056R, G2338R and G2375R), potentially invoking steric clashes with neighbouring secondary structural elements (Figure 6A–C). These glycine residues are highly conserved amongst all the plakin family members (consistency score of eight), inferring a crucial role in maintaining the structural integrity of the PRD (Figure S2). In desmoplakin PRD-A, G2056 is located at the C-terminal extremity of PR1 where the main chain executes a sharp turn (Figure 6A, left panel). Based on the desmoplakin-PRD-A^G2056R^ model, the introduction of arginine at this position is likely to substantially clash with the Nt-PR-like motif, leading to the disruption of the PRD fold (Figure 6A, right panel). This may account for the formation of insoluble PRD-A aggregates in *E. coli* and the poor expression of DSP^C^-G2056R in HeLa cells. Similarly, in desmoplakin PRD-B, G2338 and G2375 map to the ends of PR2 and PR3, respectively (Figure 6B, left panel and Figure 6C, left panel), and contribute to a conformationally critical sharp turn arrangement. An examination of the desmoplakin PRD-B models encompassing G2338R and G2375R variants reveal that R2338 may clash with PR4 (Figure 6B, right panel), whereas R2375 is likely to protrude against the N-terminal PR-like motif (Figure 6C, right panel), leading to potential structural vulnerabilities within the PRD fold. Such dramatic changes could account for the misfolding phenomenon detected for both variants following expression in bacterial cells. For other disease-associated missense mutations such as E2193K and G2647D, the substitutions may change the molecular landscape of PRs leading to energetic penalties and irreparable damage to the PRD. For example, E2193 in PRD-A mediates a salt-bridge with K2028, thereby supporting the PRD fold (Figure 6D, left panel). The modelling of desmoplakin PRD-A encompassing the E2193K mutation suggests the abolishment of this stabilising interaction. In addition, charge repulsion effects with K2028 may create additional structural vulnerabilities (Figure 6D, right panel), explaining the formation of insoluble PRD-A aggregates in *E. coli* and a poor expression of DSP^C^-E2193K in HeLa cells. Likewise, G2647 permits the close packing of H1 and H2 within the PR1 of PRD-C (Figure 6E, left panel). Incorporating D2647 at this position leads to the introduction of a negatively charged group into a predominantly non-polar environment, which may incur energetic penalties, resulting in structurally adverse repercussions (Figure 6E, right panel).

A number of disease-linked mutations (7 out of 12) were structurally tolerated insofar as they had no effect on the solubility of their respective PRD proteins when expressed in *E. coli*. We generated structural models of PRDs encompassing these mutations to examine the molecular basis underlying these effects. For two of these mutations, a positively charged residue is replaced with cysteine (R2083C and R2366C). R2083 protrudes from the H2 of PR2 of desmoplakin PRD-A and is not involved in stabilising interactions, nor forms part of the putative IF binding groove (Figure S4A, left panel). A model of desmoplakin PRD-A incorporating the C2083 variant reveals no major steric clashes (Figure S4A, right panel). In contrast, R2366 extends from the H2 of PR3 of desmoplakin PRD-B and is in close proximity to E2293 and E2290 (PR1), which could contribute to inter-helical stabilisation (Figure S4B, left panel). The introduction of C2366 at this position is predicted to lead to a loss of these interactions (Figure S4B, right panel), despite the observation that this change had no effect on the solubility of desmoplakin PRD-B in bacterial cells. For other missense disease-related mutations (K2103E and R2369Q), the substitutions provide compensatory interactions that may contribute to PRD stability. In desmoplakin PRD-A, K2103 is a solvent-exposed residue that emanates from the S2 of PR3, mediating a hydrogen bonding interaction with the hydroxyl group of S2101 (Figure S4C, left panel). The desmoplakin PRD-A model encompassing the E2103 mutation reveals a loss of this stabilising interaction. However, a compensatory ionic interaction between E2103 and K2113 may stabilise PR3 (Figure S4C, right panel). Similarly, in desmoplakin PRD-C, R2639 mediates a salt bridge interaction with D2624 (Figure S4D, left panel). This ionic interaction sets the position of the beta hairpin and is essentially conserved in each of the plakin repeats (D at position four of the PR and R/K at position 19 of the PR). Despite the predicted loss of this interaction in the desmoplakin PRD-CR2369Q model, Q2369 could form a compensatory hydrogen-bonding interaction with D2624 that pins the beta hairpin in place and stabilises the PRD (Figure S4D, right panel), thereby preventing misfolding and insolubility. Finally, the remaining missense mutations (E2343K, K2689T and R2759S) map to solvent-exposed regions and can apparently be freely substituted without serious effects on the protein stability. In the wild-type PRD-B crystal structure, E2343 projects from the S1-S2 loop of PR3 (Figure S4E, left panel). Analysis of the PRD-B model incorporating the K2343 substitution reveals that it is also solvent exposed and does not contribute to stabilising the PRD fold (Figure S4E, right panel). In desmoplakin PRD-C, K2689 protrudes from PR2 and appears to have no stabilising role (Figure S4F, left panel). The substitution of T2689 is likely to have a similar impact on the PRD structure (Figure S4F, right panel). In desmoplakin PRD-C, R2759 is a solvent exposed residue that extends from PR4 and does not contribute to stabilising the PRD fold (Figure S4G, left panel). The substitution of S2689 is unlikely to adversely impact the PRD structure (Figure S4G, right panel). This raises the possibility that the mechanism by which these mutations induce a pathological phenotype may extend beyond mere structural effects on the PRD fold.

## 3. Discussion

Desmoplakin is an essential component of desmosomes, the intercellular junctions of epithelia and cardiac muscle. It interacts with other desmosomal proteins via its N-terminal head domain and with IFs via its C-terminal tail region, consisting of three PRDs and a linker module. The interaction of desmoplakin with the cytoskeleton, through its PRDs and linker module, is vital for the maintenance of tissue integrity. The mechanism by which desmoplakin engages with IFs involves coiled-coil IF rods slotting into the basic grooves of its PRDs and linker module by means of electrostatic complementarity [5,8,9]. Desmoplakin is subject to mutations throughout its entire length, with numerous missense mutations located within the three PRDs [10]. The mechanism by which these mutations result in disease is currently unknown.

We investigated 12 disease-causing mutations identified from the HGMD database, four each in PRD-A, -B and -C. Of these, five variants (G2056R, E2193K, G2338R, G2375R and G2647D) resulted in either the complete or partial insolubility of their respective PRDs following recombinant expression in bacteria, suggesting that these mutations compromised PRD structure, leading to the formation of insoluble aggregates. The substitution of amino acids that rendered the PRD insoluble following a recombinant expression in bacteria occurs in positions that are highly conserved across plakin family members. By contrast, mutation of residues that had no effect on solubility were less well conserved within the plakin family. In the last decade, various in silico bioinformatic tools have been developed that predict the likely pathogenicity of missense variants. Of the interactive tools we used to predict the deleterious effects of the 12 missense mutations, Missense 3D demonstrated the closest correlation with our bacterial expression data, accurately assigning four of the five insolubility-inducing mutations as structurally damaging. The remaining substitutions, with the exception of E2193K, were correctly predicted using Missense 3D as structurally tolerated. With the exception of SIFT, which showed a moderate agreement with our data, the other protein stability predictors demonstrated minimal correlation with our bacterial expression results. Of course, the possibility exists that some of the missense mutations examined may introduce subtle structural vulnerabilities that are not sufficient to result in aggregate formation when expressed in bacteria.

We also assessed the impact of disease-related mutations on the ability of a truncated C-terminal desmoplakin construct to co-localise with vimentin IFs using immunofluorescence microscopy. DSP^C^ constructs encompassing the three mutations that resulted in insolubility in bacteria (i.e., G2056R, E2193K and G2375R) were very poorly expressed in HeLa cells, presumably due to destabilisation and subsequent rapid turnover. Of these, it was only possible to obtain immunofluorescence images of the DSP^C^ construct encompassing the G2375R variant, which showed compromised targeting to vimentin IFs in HeLa cells. Others have shown that mutation G2375R also adversely affects the alignment of an EGFP-DSP tail construct (consisting of EGFP fused to desmoplakin residues 2194-2871, i.e., PRD-B, the linker module, PRD-C and the GSR region) with vimentin IFs in transfected adrenal adenocarcinoma SW13 cells [32]. Thus, it would appear that the presence of PRD-A in DSP^C^ is not sufficient to alleviate the adverse effects of mutation G2375R on IF targeting. Interestingly, in patients, homozygous mutation of G2056R, heterozygous mutation of E2193K (together with a heterozygous 4bp deletion causing a frameshift at K2523 and loss of PRD-C) and homozygous mutation of G2375R all present a similar phenotype affecting the heart, skin and hair [20,23,27], emphasising the importance of the residues affected. Of the three mutations that had no effect on solubility in bacteria (R2366C, R2639Q and K2689T), none affected targeting to vimentin IFs in transfected HeLa cells. This is perhaps surprising in the case of the R2366C variant, as this mutation completely abolishes the binding of the EGFP-DSP tail protein to IF proteins including vimentin in overlay assays [33]. Thus, despite the fact that PRD-A is dispensable with respect to cytoskeletal targeting (see below), we cannot rule out the possibility that its presence in the DSP^C^-R2366C construct may have some beneficial effect. Overall, the data suggest that insolubility in bacteria correlates, to some degree, with poor expression profiles in cultured cells, most likely because of the deleterious effects on the tertiary structure and subsequent degradation, and in one case (G3275R), correlates with defective targeting to the cytoskeleton.

The basic character of PRD grooves is likely to be an important determinant of their binding affinity to IF rods [5]. Plakin protein PRD-Cs offer highly electropositive grooves (+4 to +7), PRD-Bs possess moderately basic grooves (+3 to +4) and PRD-A modules tend to have the least basic character (+1 to +3). In desmoplakin, PRD-C has seven basic residues, PRD-B encompasses four (excluding two additional positively charged side chains from the Nt-PR-like motif) and PRD-A contains four (excluding one additional positively charged side chain from the Nt-PR-like motif). This may account, at least in part, for why the targeting of truncated DSP^C^ proteins lacking PRD-A to the cytoskeleton in HeLa cells is not dramatically affected, whereas truncated DSP^C^ proteins devoid of either PRD-B or PRD-C exhibit compromised targeting. However, it is important to note that the binding affinity for IFs is unlikely to be determined solely by the basic character of the PRD groove, as basic residues bordering the groove, steric fit and nonpolar interactions may also contribute.

An intriguing possibility is that some disease-linked mutations could impact post-translational modification sites that are important in regulating desmoplakin-IF interactions [34,35]. Using the MusiteDeep server [36], we have mapped potential phosphorylation sites within desmoplakin PRDs. Interestingly, disease-linked mutations K2103E and E2193K border the predicted phosphorylation sites S2106 and S2022 in PRD-A. Additionally, potential phosphorylation sites are gained as a consequence of disease mutations K2689T and R2759S in PRD-C. Further studies will be required to characterize the functional and biochemical consequences of mutation-mediated changes to post-translational modification sites in desmoplakin.

Based upon our molecular modelling, we predict that disease-causing mutations could affect the structural properties of desmoplakin PRDs through (i) clashes with secondary structural elements (G2056R, G2338R and G2375R), (ii) the loss of intra-molecular stabilising interactions (E2193K), (iii) charge repulsion effects (E2193K) and (iv) energetic penalties as a consequence of introducing buried charges in non-polar environments (G2647D). Minimal structural changes are predicted for mutations R2083C, K2103E, E2343K, R2366C, R2639Q, K2689T and R2759S. Notably all of the substitutions examined, with the exception of G2056R, are distal to the putative IF binding site (i.e., the basic groove) and so are unlikely to directly interfere with cytoskeletal attachments. The question remains as to how mutations assigned as structurally tolerated based on our modelling exert a deleterious effect that ultimately leads to a pathological phenotype in patients. One possibility is that they cause minor perturbations in the domain structure that our in vitro assays and structural modelling are not sufficiently sensitive to detect. In the case of the R2366C variant, which is structurally tolerated despite the loss of guanidinium-mediated stabilising interactions, the thiol group of C2366 may be susceptible to oxidation, which could generate physiologically irrelevant disulphide-linked desmoplakin dimers in vivo. This mechanism could also apply to the R2083C mutation. Moreover, it is possible that the introduction of an additional negative charge moiety in the K2103E variant may compromise binding to IF rods bearing multi-acidic surfaces via electrostatic repulsion. Alternate molecular mechanisms beyond that of protein destabilisation and subsequent degradation might account for the pathogenic effects of other mutations including adaptations in ligand binding dynamics, protein trafficking and cell signalling. The challenge will be to identify potential molecular mechanisms underlying all the pathogenic mutations, which could pave the way for developing novel therapeutic strategies against ARVC and skin blistering diseases.

## 4. Materials and Methods

### 4.1. Solubility Testing of Desmoplakin PRD Mutants

DNA encoding human desmoplakin PRD-A (residues 1960-2208), PRD-B (residues 2209-2456) and PRD-C (2609-2822) were cloned into vector pProExHTc (Invitrogen, Loughborough, UK). PRD missense mutants were produced by site-directed mutagenesis using standard techniques. Overnight cultures of transformed *E. coli* cells (strain DH5α) were grown in LB medium and aliquots (0.5 mL) added to fresh LB (10 mL). The cells were grown for 2 h at 37 °C, expression was induced with 1 mM IPTG, and the cultures were grown for a further 3 h at 37 °C. Cells were harvested by centrifugation, resuspended in PBS with protease inhibitors (Roche, Welwyn Garden City, UK) (5 mL) and sonicated on ice (6 × 30 sec, maximum setting). Lysed cells were centrifuged (13,000 rpm for 5 min at 4 °C) and the soluble and insoluble fractions were analysed using SDS-PAGE. Gels were stained using Quick Coomassie stain (Generon, Slough, UK).

### 4.2. Expression of Desmoplakin Proteins in HeLa Cells, Immunofluorescence Microscopy and Western Blotting

DNA encoding a truncated human desmoplakin protein (residues 1960-2822) with a C-terminal Flag tag (DYKDDDDK) was cloned into expression vector pcDNA3.1 (−) (Life Technologies, Warrington, UK) (construct DSP^C^) (Figure S2). Missense mutants in construct DSP^C^ were generated by site-directed mutagenesis using standard techniques. DNA encoding a desmoplakin DSP^C^ΔPRD-A protein consisting of residues 2209-2822 with a C-terminal Flag tag, a DSP^C^ΔPRD-B protein consisting of residues 1960-2208 fused to residues 2454-2822 (i.e., excluding residues 2209-2453 encoding PRD-B) with a C-terminal Flag tag, and a DSP^C^ΔPRD-C protein consisting of residues 1960-2608 with a C-terminal Flag tag, was also cloned into pcDNA3.1(−). Constructs were transfected into cultured mycoplasma-free HeLa cells (obtained from Cancer Research UK, London Research Institute) (also supplied by the European Collection of Authenticated Cell Cultures) using GeneJammer transfection reagent (Agilent Technologies, Cheadle, UK) according to the manufacturer’s instructions. Cells were grown on glass coverslips in complete media for 24–36 h prior to transfection and immunofluorescence microscopy. At 48 h, following transfection, cells were fixed for 10 min in 4% paraformaldehyde and permeabilised for 2 min with 0.1% Triton X-100. Cells were co-stained with anti-Flag (Sigma-Aldrich, Gillingham, UK, F1804, 1000-fold) and anti-vimentin (Cell Signaling, London, UK, 3932, 50-fold) antibodies, followed by the appropriate AlexaFluor-conjugated secondary antibodies (Invitrogen, Loughborough, UK, A-11019 and A11070, 1000-fold). Coverslips were mounted onto microscope slides using SlowFade Gold antifade reagent (Life Technologies, Warrington, UK) and images were captured using a Zeiss LSM780 confocal microscope. Co-localisation of desmoplakin constructs and vimentin was quantified using the JACoP plugin from ImageJ (https://imagej.nih.gov/ij, accessed 1 June 2021). A set of commonly used co-localization indicators was examined by visual inspection of the staining using the decision tree proposed by the JACoP developers. Manders’ coefficient was chosen as the most appropriate method because it measures the fraction of pixels with positive values in two channels regardless of signal levels. This is important because the expression of transiently transfected proteins, and hence the signal in one channel, may vary between images. For westerns, transfected cells were lysed in SDS sample buffer, resolved using SDS-polyacrylamide gel electrophoresis and transferred to Hybond-LFP polyvinylidene difluoride membrane. Blots were probed with anti-Flag (Sigma-Aldrich, Gillingham, UK, F7425, 3000-fold) and anti-actin (Sigma-Aldrich, Gillingham, UK, A5441, 20,000-fold) antibodies, followed by the appropriate HRP-conjugated secondary antibodies (Dako, Cheadle, UK, P0448 and P0447, 1000-fold).

### 4.3. Predicting the Functional and Structural Effects of Desmoplakin PRD Variants

The potential impact of missense mutations on desmoplakin PRD function and structure was assessed using a variety of in silico tools, accessed through their online servers with default settings: SIFT (https://sift.bii.a-star.edu.sg/, accessed 1 June 2021), Missense3D (http://missense3d.bc.ic.ac.uk, accessed 1 June 2021), PolyPhen-2 (http://genetics.bwh.harvard.edu/pph2/, accessed 1 June 2021) and DynaMut (http://biosig.unimelb.edu.au/dynamut/, accessed 1 June 2021). Predictions by ENCoM, DUET and SDM were mined from the DynaMut results page, as it runs them as part of its own scoring protocol. Alignments were performed using the PRALINE multiple sequence alignment toolkit (http://zeus.few.vu.nl/programs/pralinewww/, accessed 1 June 2021).

### 4.4. Structural Modelling of Desmoplakin PRD Variants

The structures of desmoplakin PRD encompassing disease-linked variants were generated using the I-TASSER (Iterative Threading ASSEmbly Refinement) server. Briefly, the meta threading server, LOMETS2, was initially used to thread target sequences through the Protein Data Bank (PDB) library. Continuous fragments were excised from LOMETS2 alignments and reassembled using replica-exchange Monte Carlo simulations. The simulation trajectories were then clustered and used as the preliminary state for subsequent round I-TASSER assembly simulations. Finally, lowest energy molecular models were selected and refined using fragment-based molecular dynamic simulations to improve hydrogen-bonding interactions and omit steric clashes. The ranking of models was based on their I-TASSER confidence (C) score (range −5 to +2 with a higher score correlating with an improved model).

## Figures and Tables

**Table 1:** Impact of disease-associated missense mutations on expression of recombinant desmoplakin PRDs in *E. coli* and predicted effects of mutations on PRD function. Mutations in *DSP* were identified using the Human Gene Mutation Database (http://www.hgmd.cf.ac.uk/ac/index.php, accessed 1 June 2020). The impact of mutations on PRD folding was measured by assessing the solubility/insolubility of expressed PRDs in *E. coli* cells. The degree of sequence conservation or consistency score (corresponds to 0 for the least conserved alignment position and up to 10 for the most conserved alignment position) for each residue position mutated was determined by PRALINE (Figure S2). The SIFT toolkit was used to predict whether mutations are either deleterious to protein function or tolerated. The value in parentheses is the normalised probability that the amino acid change is tolerated (scores of less than 0.05 are classified as deleterious using SIFT). ^∫^ Low confidence predictions. The impact of mutations on PRD structure was predicted using Missense3D. Mutations that are predicted to caused structural damage were due to either a replacement of buried Gly (*), disallowed phi/psi angles (**) or buried charge introduced/buried Gly replaced (***). I-TASSER confidence (C-Score) refers to the quality of the molecular model (range −5 to +2 with a higher score correlating with an improved model).

PRD	Mutation	Disease	Reference	Bacterial Expression	Consistency Score	SIFT	Misense3D	I-TASSERC-Score
A	G2056R	Cardiomyopathy, skin and hair abnormalities	Christensen et al. [20]	Insoluble	8	Deleterious (0.01)	Structural damage *	1.58
A	R2083C	Long QT syndrome	Brion et al. [21]	Soluble	3	Deleterious (0.04)	No damage	1.41
A	K2103E	Cardiomyopathy	Dal Ferro et al. [22]	Soluble	4	Tolerant (0.20)	No damage	1.40
A	E2193K	Cardiomyopathy, skin and hair abnormalities	Yesudian et al. [23]	Insoluble	7	Tolerant (0.07)	No damage	1.58
B	G2338R	Cardiomyopathy	Walsh et al. [24]	Insoluble	8	Deleterious (0.00) ^∫^	Structural damage **	1.42
B	E2343K	Cardiomyopathy	Gandjbakhch et al. [25]	Soluble	4	Tolerant (0.42)	No damage	1.43
B	R2366C	Skin and hair abnormalities	Whittock et al. [26]	Soluble	6	Deleterious (0.02) ^∫^	No damage	1.42
B	G2375R	Cardiomyopathy, skin and hair abnormalities	Alcalai et al. [27]	Insoluble	8	Deleterious (0.00) ^∫^	Structural damage **	1.41
C	R2639Q	Cardiomyopathy	Yu et al. [28]	Soluble	5	Tolerant (0.06)	No damage	0.61
C	G2647D	Cardiomyopathy	Walsh et al. [24]	Soluble/Insoluble	6	Deleterious (0.01)	Structural damage ***	0.51
C	K2689T	Cardiomyopathy	Bao et al. [29]	Soluble	6	Tolerant (0.23)	No damage	0.59
C	R2759S	Cardiomyopathy	Quarta et al. [30]	Soluble	5	Tolerant (0.27)	No damage	0.53

## Data Availability

Not applicable.

## Supplementary Materials

The following supporting information can be downloaded at: https://www.mdpi.com/article/10.3390/ijms23010529/s1.
