# Supplementary material for: Missense Mutations in Desmoplakin Plakin Repeat Domains Have Dramatic Effects on Domain Structure and Function
Source: Int J Mol Sci. 2022 Jan 4;23(1):529. doi: 10.3390/ijms23010529 (PMC8745463; doi:10.3390/ijms23010529)
Supplement: Supplementary file 1 [file ijms-23-00529-s001.zip › ijms-1521384-supplementary.pdf]

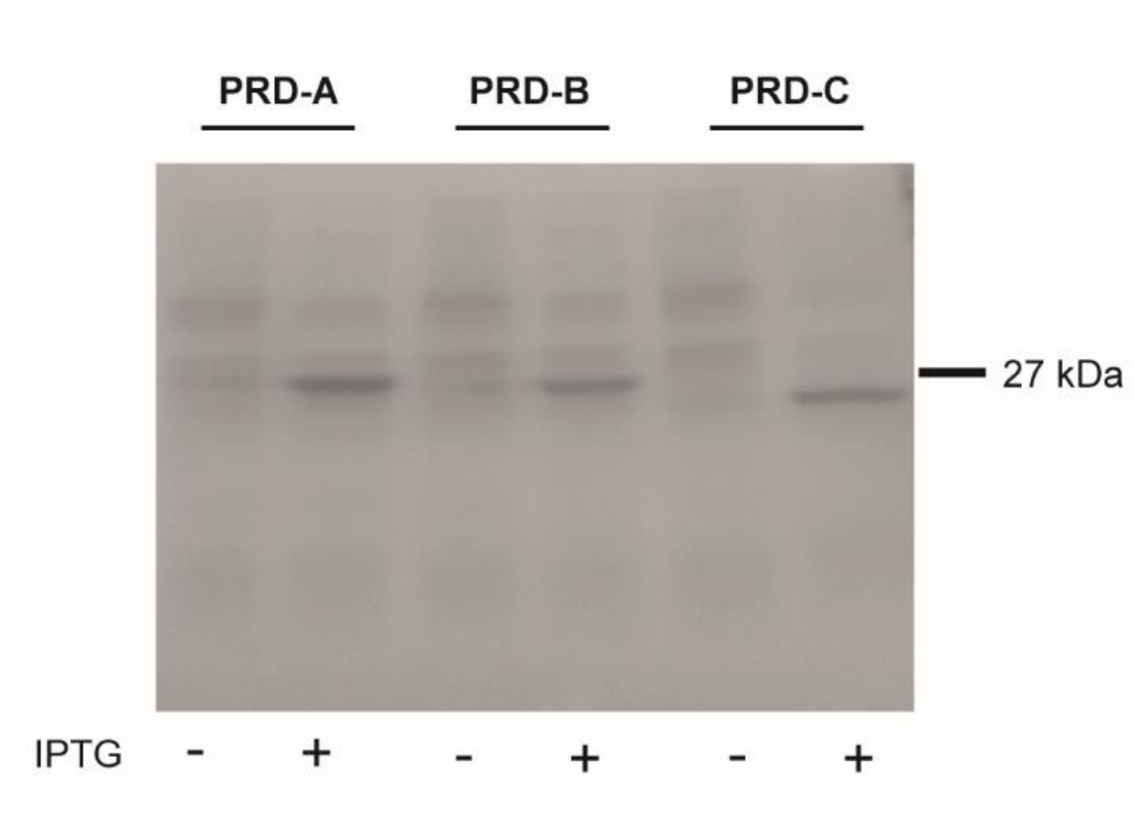

Figure S1 Induction of desmoplakin PRD expression with IPTG. Bacterial cells transformed with plasmids encoding wild-type desmoplakin PRDs A, B and C were grown for 5h, or grown for 2h, induced with 1mM-IPTG and grown for a further 3h. Cells were harvested, resuspended in SDS-PAGE sample buffer and proteins resolved by SDS-PAGE and stained with Coomassie.

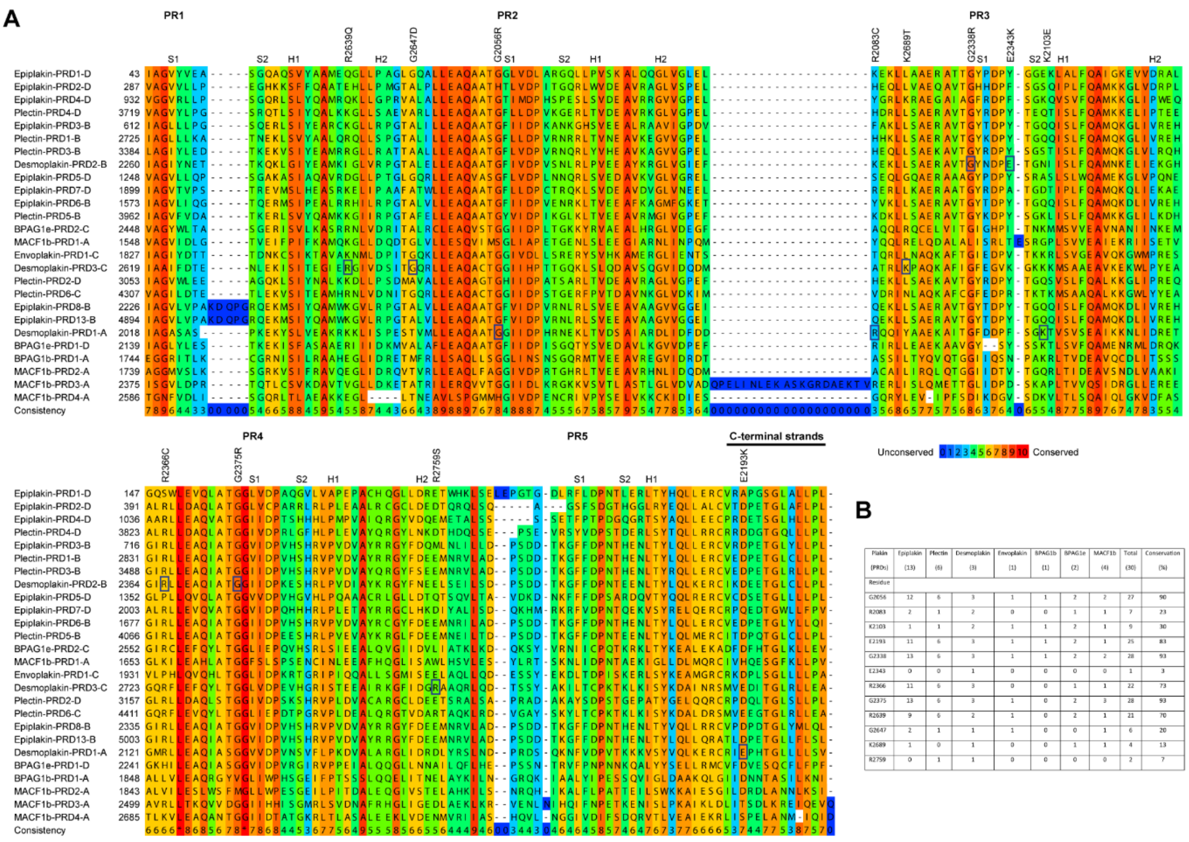

Plakin (PRDs)	Epiplakin (13)	Plectin (6)	Desmoplakin (3)	Envoplakin (1)	BPAG1b (1)	BPAG1e (2)	MACF1b (4)	Total (30)	Conservation (%)
Residue									
G2056	12	6	3	1	1	2	2	27	90
R2083	2	1	2	0	0	1	1	7	23
K2103	1	1	2	1	1	2	1	9	30
E2193	11	6	3	1	1	2	1	25	83
G2338	13	6	3	1	1	2	2	28	93
E2343	0	0	1	0	0	0	0	1	3
R2366	11	6	3	0	0	1	1	22	73
G2375	13	6	3	1	0	2	3	28	93
R2639	9	6	2	1	0	2	1	21	70
G2647	2	1	1	1	0	0	1	6	20
K2689	1	0	1	0	0	1	1	4	13
R2759	0	1	1	0	0	0	0	2	7

Figure S2 A) Sequence alignment of plakin repeat domains. Sequences were obtained from Uniprot (accession numbers O60437 (periplakin), P15924 (desmoplakin), Q92817 (periplakin), Q15149-2 (plectin), Q03001-3 (BPAG1e), Q9UPN3-1 (MACF1b) and Q03001-7 (BPAG1b). Epiplakin PRDs 9-12 have been omitted as they are identical to PRD8. Secondary structure for the plakin repeat motif is shown (S1, strand1; S2, strand2: H1, helix1; H2, helix2). Alignment was performed using the PRALINE multiple sequence alignment toolkit. The colour scheme shows the degree of amino acid conservation. The consistency score corresponds to the degree of sequence conservation (0 for the least conserved alignment position and 10 for the most conserved alignment position). Boxes indicate the position of the mutations studied. Figure adapted from [1]. B) Conservation analysis of residues characterised in this study across the 30 mammalian plakin family PRDs (Epiplakin (13); Plectin (6); Desmoplakin (3); Envoplakin (1); BPAG1b (1); BPAG1e (2) and MACF1b (4)). The number of PRDs in each protein is indicated in parentheses. The number of times each residue is present within the different PRDs is listed. For the purposes of this analysis changes from arginine to lysine (at positions 2083, 2366, 2639 and 2759), lysine to arginine (at positions 2103 and 2689) and glutamic acid to aspartic acid (at positions 2193 and 2343) were not

considered to involve a loss of conservation. Hence mutation of residues that render their respective PRDs entirely insoluble in bacteria (G2056R, E2193K, G2338R and G2375R) are highly conserved (≥ 90%). Mutation G2647D renders PRD-C partially insoluble in bacteria. The glycine at position 2647 is conserved in only 20% of PRDs. However this position is occupied by alanine in the majority of other PRDs and the glycine/alanine combination is conserved in 87% of PRDs. This figure is broadly in agreement with consistency scores from PRALINE (i.e. lower than that of G2056R, G2338R and G2375R, comparable to that of E2193K, and higher than that of those residues that when mutated had no effect on the solubility of their respective PRDs when expressed in bacterial cells).

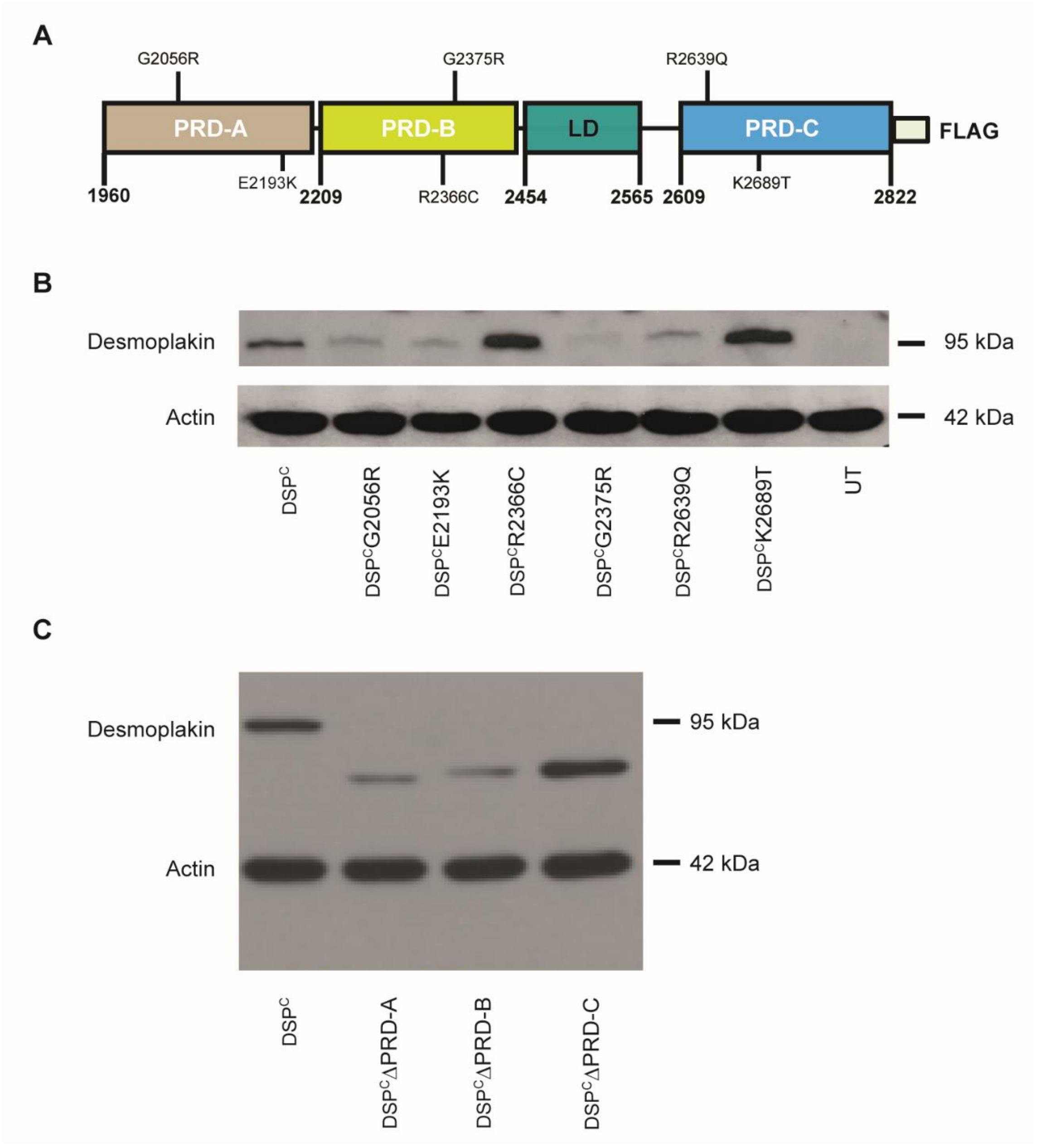

Figure S3 A) Desmoplakin construct DSP^C used to transfect HeLa cells. The positions of disease-causing mutations introduced into wild-type DSP^C are indicated. B) Western blot showing expression of wild-type and missense mutant DSP^C proteins in transfected HeLa cells. C) Western blot showing expression of wild-type and deletion mutant DSP^C proteins in transfected HeLa cells. Desmoplakin expression was detected using an anti-FLAG antibody.

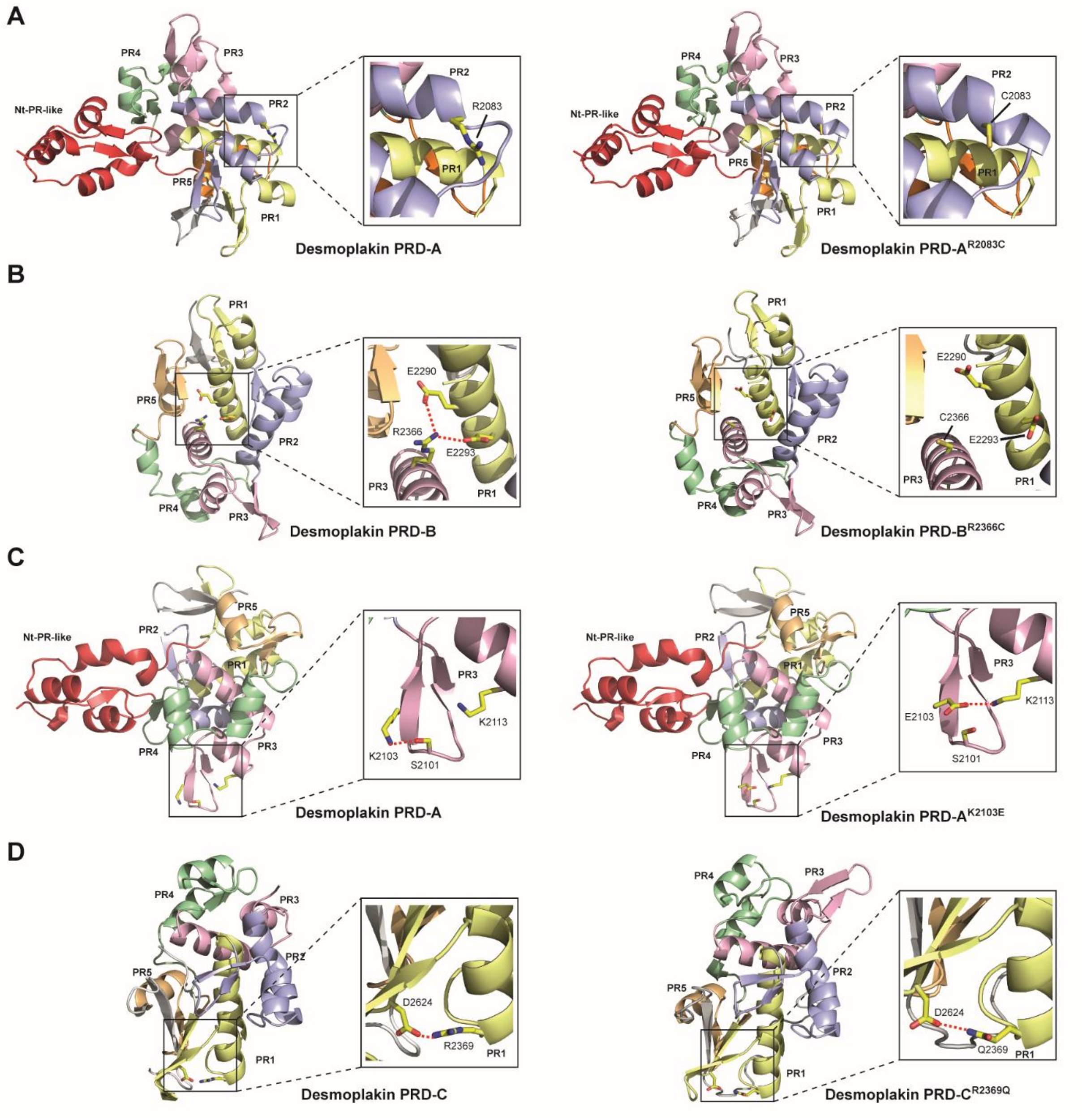
A
Nt-PR-like
PR4
PR3
PR2
PR5
PR1
R2083
Desmoplakin PRD-A
C2083
Desmoplakin PRD-A^{R2083C}
B
E2290
R2366
E2293
Desmoplakin PRD-B
C2366
Desmoplakin PRD-B^{R2366C}
C
K2113
K2103
S2101
E2103
Desmoplakin PRD-A^{K2103E}
D
D2624
R2369
Desmoplakin PRD-C
Q2369
Desmoplakin PRD-C^{R2369Q}

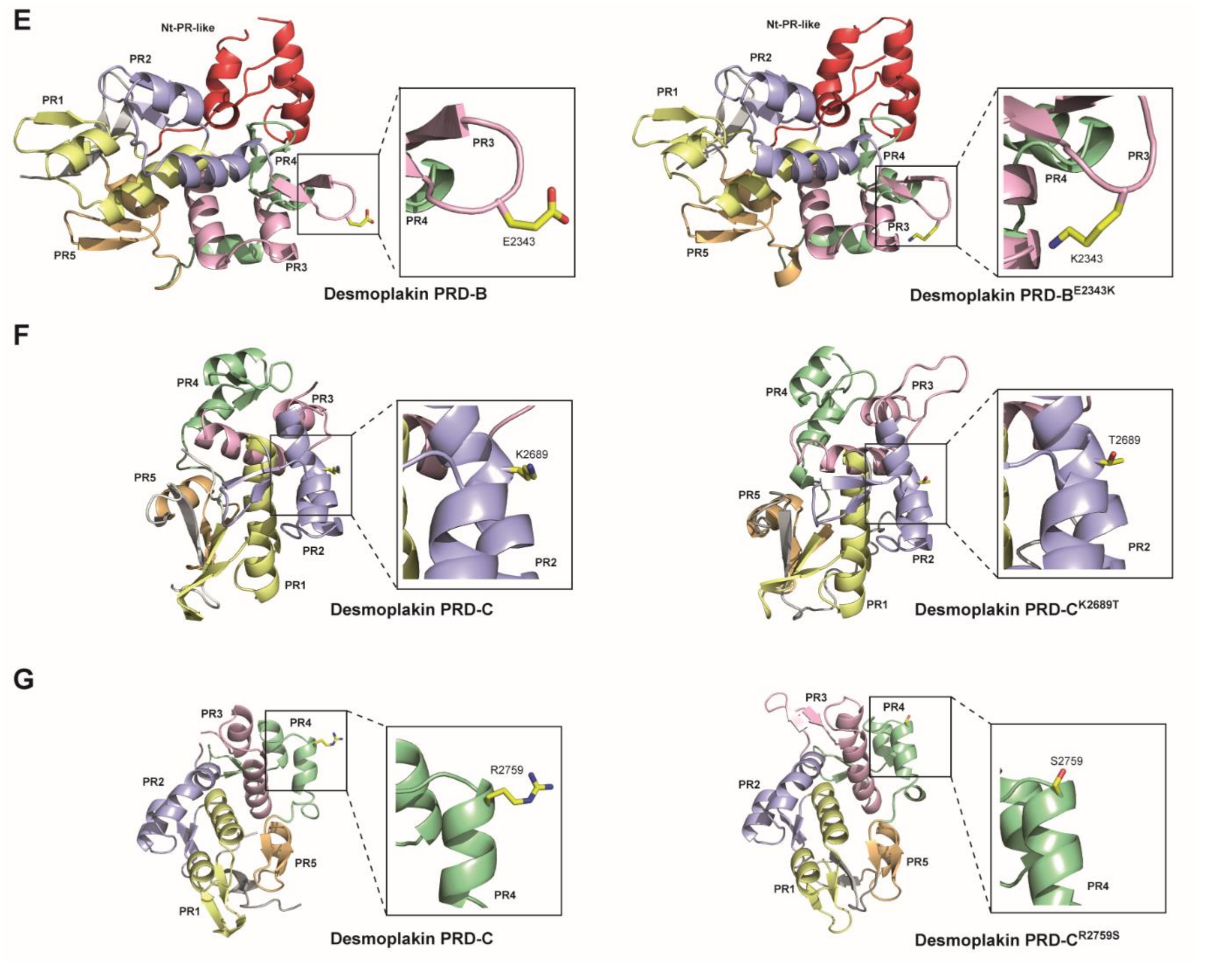

Figure S4 Modelling the effects of disease-associated missense mutations on desmoplakin PRD structure. Disease-linked variants that led to soluble desmoplakin PRD expression in bacterial cells were modelled. A) Analysing the impact of the disease-related variant R2083C on desmoplakin PRD-A. Ribbon diagram of the desmoplakin PRD-A crystal structure shows that R2083 maps to PR2 and is solvent exposed (left panel). Introduction of C2083 at this position leads to no major clashes (right panel). B) Analysing the impact of the disease-related variant R2366C on desmoplakin PRD-B. Ribbon diagram of the desmoplakin PRD-B crystal structure shows that R2366 mediates multiple ionic interactions with negatively charged residues (left panel). Introduction of C2366 leads to a loss of these interactions (right panel). C) Assessing the effect of ARVC-linked variant K2103E on desmoplakin PRD-A. Ribbon diagram of the desmoplakin PRD-A crystal structure highlighting the hydrogen-bonding

interaction (red dashed line) between K2103 and S2101 (left panel). Introduction of E2103 leads to a compensatory salt-bridge stabilising interaction with K2113 (right panel). D) Assessing the effect of ARVC-linked variant R2369Q on desmoplakin PRD-C. Ribbon diagram of the desmoplakin PRD-C crystal structure highlights that R2369 mediates salt bridge interaction with D2624 (left panel). Introduction of Q2369 abolishes this interaction but there is a possibility of a compensatory hydrogen bonding interaction with D2624 (right panel). E) Probing the impact of disease-associated variant E2343K on desmoplakin PRD-B. Ribbon diagram of the desmoplakin PRD-B crystal structure shows that E2343 is solvent exposed residue projecting from the S1-S2 loop of PR3 (left panel). Substitution of K2338 at this position leads to no substantial clashes (right panel). F) Probing the impact of disease-associated variant K2689T on desmoplakin PRD-C. Ribbon diagram of the desmoplakin PRD-C crystal structure shows that K2689 is solvent exposed residue that protrudes from PR2 (left panel). Substitution of threonine at this position leads to no substantial clashes (right panel). G) Assessing the effect of disease-linked variant R2759S on desmoplakin PRD-C. Ribbon diagram of the desmoplakin PRD-C crystal structure shows that R2759 is solvent exposed residue that projects from PR4 (left panel). Substitution of serine at this position leads to no substantial clashes (right panel). Plakin repeats 1-5 are yellow, blue, pink, green and orange, respectively, the Nt PR like domain is highlighted in red and non-repeat regions are shown in grey. Boxes show close up views of the relevant interactions.

PRD	Mutation	Bacterial Expression	PolyPren-2	DynaMut	ENCoM	mCSM	SDM	DUET
A	G2056R	Insoluble	Probably damaging (1)	Destabilising (-0.458)	Stabilising (0.678)	Destabilising (-0.83)	Destabilising (-3.24)	Destabilising (-1.157)
A	R2083C	Soluble	Probably damaging (1)	Destabilising (-0.721)	Destabilising (-0.298)	Destabilising (-1.648)	Destabilising (-0.32)	Destabilising (-1.506)
A	K2103E	Soluble	Possibly damaging (0.86)	Stabilising (0.026)	Destabilising (0.009)	Stabilising (0.103)	Stabilising (0.53)	Stabilising (0.493)
A	E2193K	Insoluble	Possibly damaging (0.95)	Stabilising (0.228)	Destabilising (0.446)	Destabilising (-0. 809)	Destabilising (-0.22)	Destabilising (-0.575)
B	G2338R	Insoluble	Probably damaging (1)	Stabilising (1.183)	Stabilising (1.390)	Destabilising (-0.868)	Destabilising (-4.04)	Destabilising (-1.173)
B	E2343K	Soluble	Possibly damaging (0.86)	Stabilising (0.922)	Destabilising (0.077)	Stabilising (0.426)	Destabilising (-0.25)	Stabilising (0.603)
B	R2366C	Soluble	Probably damaging (1)	Destabilising (-1.109)	Destabilising (-0.597)	Destabilising (-1.624)	Destabilising (-0.5)	Destabilising (-1.543)
B	G2375R	Insoluble	Probably damaging (1)	Stabilising (1.127)	Stabilising (1.120)	Destabilising (-0.603)	Destabilising (-2.09)	Destabilising (-0.786)
C	R2639Q	Soluble	Probably damaging (1)	Stabilising (0.046)	Destabilising (-0.618)	Destabilising (-0.288)	Destabilising (-0.65)	Destabilising (-0.331)
C	G2647D	Soluble/Insoluble	Probably damaging (0.98)	Destabilising (-1.254)	Destabilising (0.33)	Destabilising (-2.573)	Destabilising (-2.44)	Destabilising (-2.803)
C	K2689T	Soluble	Probably damaging (1)	Destabilising (-0.636)	Destabilising (-0.159)	Destabilising (-1.043)	Destabilising (-1.51)	Destabilising (-1.131)
C	R2759S	Soluble	Probably damaging (1)	Stabilising (0.128)	Destabilising (0.019)	Destabilising (-0.17)	Destabilising (-1.36)	Destabilising (-0.27)

Table S1 Predicted effects of disease-associated missense mutations in PRDs. The damage induced by the mutations (as measured by the solubility/insolubility of expressed PRDs in bacterial cells) was compared to structural changes predicted by PolyPhen-2, DynaMut, ENCoM, mCSM, SDM and DUET. PolyPhen-2 classifies mutations as either benign, possibly damaging or probably damaging and provides a probability score (mutations with values closer to 1 are likely to be structurally deleterious). DynaMut, ENCoM, mCSM, SDM and DUET all predict the effect of single amino acid mutations on protein stability, label mutations as destabilising or stabilising and provide a predicted change in stability. Probability scores from PolyPhen-2 and predicted changes in stability (ΔΔG in kcal/mol) from DynaMut, ENCoM, mCSM, SDM and DUET are given in parentheses. Negative ΔΔG values (and in some cases positive values close to zero) are destabilising

References

[1] F. Mohammed, C. Trieber, M. Overduin, M. Chidgey, Molecular mechanism of intermediate filament recognition by plakin proteins, Biochim Biophys Acta Mol Cell Res, 1867 (2020) 118801.
